# Advancements in Viral Genomics: Gated Recurrent Unit Modeling of SARS-CoV-2, SARS, MERS, and Ebola viruses

**DOI:** 10.1590/0037-8682-0178-2024

**Published:** 2025-02-07

**Authors:** Abhishak Raj Devaraj, Victor Jose Marianthiran

**Affiliations:** 1 Noorul Islam Centre for Higher Education, Department of Computer Applications, Tamilnadu, India.; 2 Vel Tech Multi Tech Dr. Rangarajan. Sakunthala Engineering College, Department of Artificial Intelligence and Data Science, Tamilnadu, India.

**Keywords:** Genome sequencing, Gated Recurrent Unit, Virus, Nucleotides, Deep learning

## Abstract

**Background:**

Emerging infections have posed persistent threats to humanity throughout history. Rapid and unprecedented anthropogenic, behavioral, and social transformations witnessed in the past century have expedited the emergence of novel pathogens, intensifying their impact on the global human population.

**Methods:**

This study aimed to comprehensively analyze and compare the genomic sequences of four distinct viruses: SARS-CoV-2, SARS, MERS, and Ebola. Advanced genomic sequencing techniques and a Gated Recurrent Unit-based deep learning model were used to examine the intricate genetic makeup of these viruses. The proposed study sheds light on their evolutionary dynamics, transmission patterns, and pathogenicity and contributes to the development of effective diagnostic and therapeutic interventions.

**Results:**

This model exhibited exceptional performance as evidenced by accuracy values of 99.01%, 98.91%, 98.35%, and 98.04% for SARS-CoV-2, SARS, MERS, and Ebola respectively. Precision values ranged from 98.1% to 98.72%, recall values consistently surpassed 92%, and F1 scores ranged from 95.47% to 96.37%.

**Conclusions:**

These results underscore the robustness of this model and its potential utility in genomic analysis, paving the way for enhanced understanding, preparedness, and response to emerging viral threats. In the future, this research will focus on creating better diagnostic instruments for the early identification of viral illnesses, developing vaccinations, and tailoring treatments based on the genetic composition and evolutionary patterns of different viruses. This model can be modified to examine a more extensive variety of diseases and recently discovered viruses to predict future outbreaks and their effects on global health.

## INTRODUCTION

In today’s highly populated and internationally networked world, the spread of pathogens is higher than ever before. Infectious disease pandemics have increased in frequency and complexity since 2000, owing to rapid changes, increased urbanization, and increased travel worldwide. Notable examples of these include Zika, Ebola, Nipah, severe acute respiratory syndrome (SARS), middle east respiratory syndrome (MERS), and influenza, a subtype of H5N1^1^. The SARS epidemic of 2003 was the first global public health emergency in the 21^st^ century. The H5N1 outbreak in 2005-2006 followed. The H1N1 influenza virus pandemic (2009)^2^, comeback of wild poliovirus (2014)^3^, West Africa Ebola virus epidemic (2014)^4^, Zika virus outbreak (2018)^5^, and severe acute respiratory syndrome coronavirus disease-2 (SARS-CoV-2) (2020)^6^ have all been declared public health emergencies of international concern by the World Health Organization^7^.

The risks of previously identified viruses are still unexpected and their spread is difficult to manage, similar to the recent coronavirus disease 2019 (COVID-19) and Ebola outbreaks^8^. Sequencing of most RNA viral genomes requires accurate identification of the composition of the RNA molecule. Reverse transcription PCR (RT-PCR) is an essential technique in which RNA is reverse transcribed to cDNA and can be sequenced^9^. Contemporary DNA sequencing has accelerated genome sequencing, allowing experts to decipher viral RNA genetic codes^10^.

To understand the virus and countermeasures, its makeup, function, and evolutionary tendencies must be studied. Such data also help to identify targets for effective vaccines and antivirals^11^. In this ongoing war against viruses, viral genome sequencing has proven valuable. It provides vital information about the virus’s disease-causing capabilities, transmission, and drug-resistant variations^12^ ^,^ ^13^. Therefore, the primary objective of DNA sequencing is to develop genomic therapeutics^14^. These medications seek to understand genetic differences to decipher the intricacies of illnesses and their therapies^15^. Significant efforts are being made to integrate genomic data from sequencing, population-level data, and behaviors to thoroughly investigate the connection between genetics and health. Genomic studies are crucial for understanding the development, dissemination dynamics, and potential health impacts of emerging viral variants such as SARS, Ebola, SARS-CoV-2, and MERS^16^. Integrating artificial intelligence (AI) and associated innovations, including machine learning, data analysis, and deep learning (DL), may significantly accelerate the extraction of practical insights, thereby improving global adaptability^17^ ^,^ ^18^. The increasing need for computational methods capable of analyzing large, complex, and high-dimensional genetic datasets highlights the need for accurate criteria and unique interests across the genomic data pipeline^19^. When used carefully, AI can reveal new findings regarding such datasets, provided there is an explicit set of requirements and goals to be met at different phases of the genetic data assessment process^20^.

Despite ongoing progress in genomics, a substantial portion of AI applications in this field remain in the research phase. The objectives of this study drew inspiration from the aforementioned discourse and the evolving landscape of AI in genomic sequencing. This study primarily aimed to introduce a DL-based methodology for analyzing genomic sequences related to SARS-CoV-2, Ebola, SARS, and MERS as well as investigate the associations among various genome sequences.

In viral genomics, cutting-edge computational models have dramatically changed how researchers perceive genetic changes in viruses and how they cause diseases and spread. Among these models, the Gated Recurrent Unit (GRU) is a potent assistant for studying genomic sequences, because it can capture dependencies to access long-range details and temporal features from sequential data^21^ ^,^ ^22^. GRU modeling has provided important information on several widespread viruses like SARS-CoV-2, SARS, MERS, and Ebola. This method can be applied to distinguish variants of SARS-CoV-2, SARS, MERS and Ebola viruses. GRUs excel at processing sequential data and identifying long-range dependencies, making them ideal for analyzing viral genomic sequences containing complex patterns that define different strains or variants of a virus. GRU models can be trained to detect variants, track evolutionary changes, and even predict the functional impacts of mutations, thus providing critical insights into viral behavior and aiding in pandemic monitoring efforts, as well as investigate associations between different viral genome sequences. This includes detecting evolutionary relationships, understanding recombination events, and identifying shared mutations among related viruses. Therefore, the proposed model has the ability to identify variants from genomes or genome fragments. While showcasing promising predictive results, one limitation of DL models involves the potential dependence on available genomic data, which may still need to capture emerging variants to be reported globally.

## METHODS

**FIGURE 1:**
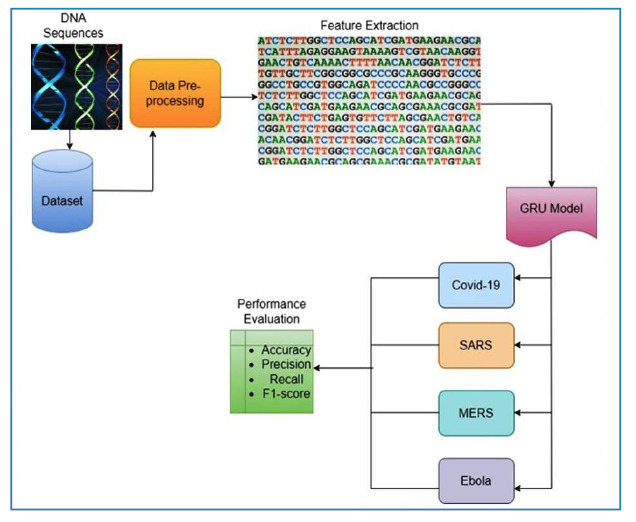
Workflow of the proposed model.

Extensive analysis of the genetic makeup and evolutionary background of viruses depends primarily on genome sequence data. The worldwide effects of the SARS-CoV-2 pandemic have increased the need for genomic sequence analysis. The genome contains complex information regarding the evolution, transmission dynamics, and virulence of a virus. The availability of genomic sequencing data has led to the development of a wide range of computational tools and methods. The GRU algorithm is a DL method that works well with time-series data, particularly genomic sequences. When applied to the study of genomic data, the GRU approach makes it easier to classify viruses according to their genetic sequences, which helps identify similarities and differences. The intricate details of the genetic analysis procedure are shown visually in Figure 1.

**Dataset:** The research dataset included metadata from GenBank downloaded on March 4, 2024, from a well-known publicly available nucleotide sequencing database supported by the National Center for Biotechnology Information. The length of each DNA sequence (base pairs) under investigation included 1920 instances of SARS (SARS COV-1): 29,751; MERS: 30,119; SARS COV-2: 29,903, and Ebola: 18,959. 

**Preprocessing:** Data preprocessing, which involves ensuring data relevance and suitability for feeding into the GRU system, is a crucial stage in the investigation procedure following data collection. To adequately prepare genetic information for assessment in this study, many sequential procedures must be performed. The tasks involved in this process included normalizing the data, eliminating repetitive data sequences, discarding non-coding sections, standardizing the length of the patterns, and transforming the sequencing data into a format suited to the GRU approach. Eliminating duplicate and non-coding sequences removes possible biases and reduces the collection size. Furthermore, normalization guarantees that the values of each feature are confined to a uniform range, leading to a greater accuracy of the following DL classifier.

Given that the viruses being examined belong to the RNA category and that the input information in the FASTA design contains DNA, preprocessing of sequences that transforms the DNA into RNA is necessary. This change is necessary for RNA viruses to use uracil (U) instead of thymine (T) to make up all nucleotides. For instance, the DNA pattern ATTAAAGGTT served as the starting point for generating .fna file. Given that viral genomes are composed of RNA, we employed a transcription strategy that substituted thymine (T) with uracil (U) throughout the sequence. This change resulted in an amino acid sequence in the RNA that initiated with AUUAAAGGUU. The transcribe() function is crucial for converting DNA to mRNA and ensuring the accuracy of subsequent analyses, including translation through the amino acid sequence.

**Feature Extraction:** The Sequence data are converted into numerical characteristics that can be easily used by GRU systems operating in both directions. Feature extraction helps to identify specific parts of the genome that are important and associated with specific biological features or outcomes, whereas GRU processes complete genomic sequences and searches genomic sequences of bacteria for specific patterns that are unique for each bacterium. These features include GC content, codon bias, nucleotide arrangement, protein sequence, structural information, amino acid sequence, secondary structure information, and protein regions. These efficient extractions are necessary for ensuring the quality of the dataset and improving the discrimination of the DL models.

**DL Classifier:** The GRU is a type of recurrent neural network (RNN) architecture that addresses some of the limitations of traditional RNNs, particularly the vanishing gradient problem, which hinders the network’s ability to capture long-term dependencies in sequential data. In its structure, a GRU unit comprises of two essential components: an update gate (*z_t*) and a reset gate (*r_t*). Systems require gateways to regulate information delivery. From the quantity to be eliminated, they decide how much of the previous disguised phase to maintain. The update gate determines which past-state data are to be maintained, whereas the reset gate is discarded. Every input element is analyzed separately using the GRU algorithm, as shown in Algorithm 1. The system calculates the reset gate, update gate, and Candidate Hidden State ((*h_t* ) ˇ ) at each time step. This new prospective concealed phase is computed using the input and reset gates. The GRU changes the ultimate hidden state (*h_t*) to maintain essential details while processing sequential inputs by executing the aforementioned calculations.

**Algorithm 1: GRU**

***Input: Genomic sequences of four distinct viruses: COVID-19, SARS, MERS and Ebola.***

***Output: Genome Sequence Analysis of four distinct viruses: COVID-19, SARS, MERS and Ebola***

**Begin**

- Data Preprocessing:
- Generate numerical representations from genomic sequences.
- Convert DNA sequence to RNA
- Split the dataset into training and testing sets.

**Feature Extraction:**

- Features such as GC content, codon bias, nucleotide patterns, protein sequences, structural data, amino acid arrangement and protein domains are extracted.

**Define GRU Model:**

( Initialize parameters:

w_i, b_i, u_i (Input Gate)

w_f, b_f, u_f (Forward Gate)

w_o, b_o, u_o (Output Gate)

w_c, b_c, u_c (Cell Update)

( Define the forward pass equation-

*Update Gate:* $i_{-}t=\sigma \left(W_{-}i*x_{-}t+U_{-}i*h_{-}(t-1)+b_{-}i\right)$

*Forget Gate:* $f_{-}t=\sigma \left(W_{-}f*x_{-}t+U_{-}f*h_{-}(t-1)+b_{-}f\right)$

*Output Gate:* $o_{-}t=\sigma \left(W_{-}o*x_{-}t+U_{-}o*h_{-}(t-1)+b_{-}o\right)$

*Cell Update:* $c_{-}t=tanh\left(W_{-}c*x_{-}t+U_{-}c*\left(r_{-}t*h_{-}(t-1)\right)+b_{-}c\right)$

*Hidden State:* $h_{-}t=\left(1-i_{-}t\right)*c_{-}t+i_{-}t*h_{-}(t-1)$

*Output:* $y_{-}t=h_{-}t$

**Training:**

- Define loss function (categorical cross entropy)
- Iterate over epochs, changing the parameters to reduce the loss

**Evaluation:**

- Evaluate the learned model using the testing dataset.
- Analyse performance parameters (such as accuracy, precision, recall and F1-score)
- Analyze the results of the model concerning genetic relationships.
- Identify pertinent data, including alignment trends and similarities.
- Create an understanding of the relationships between various genomic sequences.

**End**

The input at current instant is represented by *x_t*, the update and reset gates are denoted by *Z_t* and *r_t*, respectively and the outputs at current and prior instants are *h_t* and *h_(t-1)*. The GRU parameters are calculated as follows:

Z_t=σ(W_z.[h_(t-1),x_t ]) (1)

r_t=σ(W_r.[h_(t-1),x_t ]) (2)

(h_t ) ˇ=tanh(W.[r_t*h_(t-1),x_t ]) (3)

h_t=(1-Z_t )*h_(t-1)+Z_t*(h_t ) ˇ (4)

where the parameter matrices are *W_z, W* and *W_r*, the candidate hidden state is *(h_t ) ˇ*, and the sigmoid and tanh activation functions are denoted by *σ(x)* and *tanh(x)*.

sigmoid=1/(1+e^(-x) ) (5)

tanh=(e^x-e^(-x))/(e^x+e^(-x) ) (6)

In the proposed study, GRUs typically encoded the viral genome sequence into a numerical representation that can be fed into the GRU network. Each nucleotide in a genome sequence is typically represented using a one-hot encoding scheme, converting it into a vector, where each element corresponds to a specific nucleotide (A, C, G, or T). These one-hot encoded vectors were then sequentially fed into the GRU network, allowing the model to learn patterns and dependencies within the viral genome sequence.

One key advantage of the GRU in this study is its ability to capture both short- and long-term dependencies within the genome sequence. This is crucial for understanding various aspects of viral behavior such as protein-coding regions, regulatory elements, and evolutionary patterns. GRUs accomplish this by utilizing gating mechanisms that control the flow of information through networks. These gates (update and reset gates) enable the model to selectively update its internal state based on current and previous inputs.

**Model optimization and hyper-parameter:** deep neural network hyperparameters are determined empirically and have a significant impact on learning. Consequently, various variables are tested to determine the best classification performance. 

**Evaluation Metrics:** Evaluation metrics offer a numerical representation of a model’s performance, enabling a methodical and impartial researcher assessment of its efficacy. 

## RESULTS

Coding investigations were conducted using Google Colab Notebook with the Bio-Python programming language as a seamless gateway. The recommended model was implemented by leveraging only the functionalities provided by Scikit-learn and Keras, which enabled efficient data preprocessing. The computer platform utilized for the algorithm development was equipped with 8 GB of RAM and an 1.6 GHz Intel Core i5 CPU. The computational framework facilitated learning and model optimization by implementing the training techniques with resilience. The seamless incorporation of the Google Colab, Bio-Python, Scikit-learn, and Keras libraries significantly prompted our research objectives. This collaborative partnership facilitated a more streamlined and influential method for analyzing genetic data.

**FIGURE 2:**
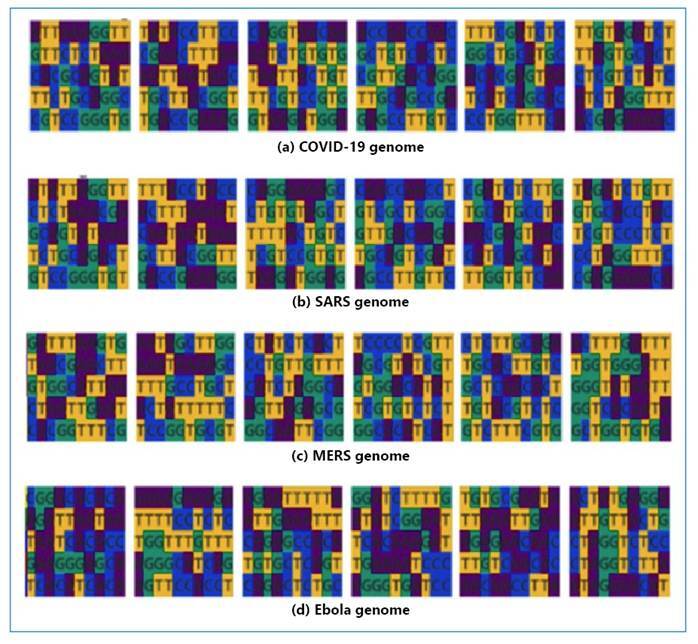
Visualization of nucleotides in viral DNA sequences.

Every virus exhibits distinct characteristics, which are determined by its precise sequence and nucleotide content. The order of nucleotide sequences is essential for understanding the relationship between the virus and the host organism and transmission among humans. The complex interrelationships underlying the DNA structure of a gene as well as the associated amino acid sequence were accurately regulated by the genetic algorithm. Figure 2 shows a thorough visualization of each DNA genome series, revealing the unique genetic content of each pathogen. This study elucidated the intricate interplay between viral proteins in the host system and unveils their fundamental chemical mechanics.

**FIGURE 3:**
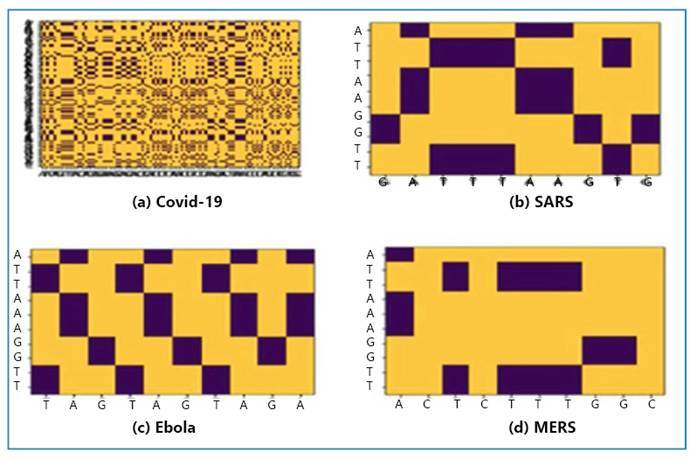
Dot plot of each virus.

Dot plots are invaluable for analyzing specific sections or whole genomes of two pathogens. Figure 3 shows a dot plot for each virus. Sequence alignment was performed using both axes, with dots from the panel indicating the locations where nucleotides from the two identical sequences overlapped. A dot appeared when the nucleotides were the same; otherwise, no variations occurred. Because minimal discernible differences were observed across the analyzed sequences, the result exhibited a high level of comparison, as evidenced by the presence of a cross-section of points. Dot plots provided a visual depiction of the genetic distinctions and parallels among the genetic materials of the MERS, SARS-CoV-2, SARS, and Ebola viruses used for comparison. Understanding the interconnections between these viruses is necessary to develop effective treatments and preventive measures. The complex structures shown in the dot plots offered significant insights into the molecular intricacies that distinguish the studied viruses, thereby assisting in understanding the development, spread, and harmfulness of these infectious agents.

**FIGURE 4:**
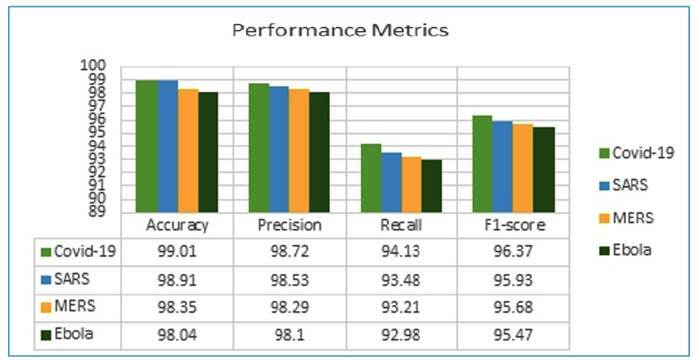
Performance comparison of different viruses.

Figure 4 shows the results of categorizing all the viral genes using the GRU model, which was crucial for evaluating the models. The 99.01% accuracy of the model allowed the SARS-CoV-2 version to recognize circumstances accurately. The low false positive rate for each infection was supported by consistently high accuracy ratings of 98.1%-98.72%. The recall ratings of 92.98%-94.13% of the models demonstrated their accuracy in recognizing actual instances. Despite balancing accuracy and memory, an F1-score of 95.47%-96.37% suggested excellent viral protection. The GRU viral gene classification method was resilient and effective, as shown in Table 1.

**TABLE 1:** Performance comparison of the proposed methodology with existing literature.

Sl. No:	Infection	Dataset	Methodology	Accuracy (%)	References
1)	COVID-19	GenBank	KNN	98.89	^26^
2)	SARS	GenBank	LSTM	97.52	^27^
3)	SARS	GenBank	Neuro chaos Learning	99.8	^28^
4)	MERS	GenBank	CNN+LSTM	99.27	^29^
5)	MERS	GenBank	CNN	93	^30^
6)	EBOLA	GenBank	EdeepVPP	91	^32^
7)	COVID-19	GenBank	GRU	99.01	Proposed
8)	SARS	GenBank	GRU	98.91	Proposed
9)	MERS	GenBank	GRU	98.35	Proposed
10)	EBOLA	GenBank	GRU	98.04	Proposed

Phylogenetic analysis was performed to investigate the molecular characteristics and dynamics of viral evolution using GRU networks to detect mutations that alter virulence, host tropism, and transmission efficiency. In addition, the study also helped learn about the SARS and MERS epidemics and inspired the development of new diagnostic tools and treatments, thus limiting their spread to the global public health system. Using GRU networks to dissect and compare Ebola virus genomes from their sources in human patients and the wildlife reservoir, researchers have tracked the spread of the virus into distinct regions and mapped hotspots where transmission is likely to occur. Additionally, GRU-based predictive models have also had implications in predicting the course that epidemics are expected to follow, making it easier to initiate early detection and intervention efforts that will ensure that the spread of the virus is curbed and the number of lives lost is minimized. The implementation of GRU modeling in viral genomics has made tremendous progress regarding viral evolution, transmission, and host-pathogen interactions. By accounting for the temporal context surrounding genomic sequences, GRU networks enable researchers to extract relevant information from large-scale datasets of viral genomes to inform public health sector responses and strategies for disease control and prevention. In the face of new infection threats, integrative approaches combining computational and experimental techniques, as well as GRU modeling, promise considerable advantages against viral outbreaks on a global scale.

## DISCUSSION

This research has paved the way for extensive therapeutic advancements owing to the valuable insights provided by analyzing SARS, SARS-CoV-2, Ebola, and MERS through genomic studies, including sequence alignment, dot plot analysis, and the GRU methodology. The SARS-CoV-2 classification algorithm has demonstrated a remarkable performance and accuracy in SARS-CoV-2 classification, as high as 99%. The results obtained were less than 1% for the SD and CE comparing the distinct viral genomes, which suggests the effectiveness of these methods^23^ ^,^ ^24^ ^,^ ^25^. This degree of classification is essential for an accurate and timely diagnosis of the problem and for enhancing the efficiency of dealing with an illness. The identification of unique genetic associations related to viral susceptibility, transmissibility, and therapeutic resistance will shape future research on viral pathobiology and transmission^26^. Different techniques can provide more focused therapeutic strategies that may enhance the possibility of treating various diseases at a higher level^27^ ^,^ ^28^ ^,^ ^29^. This study sets the platform for the development of specific drugs and effective diagnostic methods that are vital in managing the outcomes of new diseases as they emerge, thereby improving the quality of patient care. This promise will be realized through the identification of potential therapeutic goals.

This study also showed the importance of sequencing viral genomes to predict and assess threats emerging from viral diseases. Using identifiers that work on behavioral genomic surveillance, researchers can monitor new variations that may evolve and change the approaches employed in treatment plans^30^. One of the novelties of this study is the application of computational methods that allow for more effective evaluation of genomic data and the identification of reasonable conclusions. One of the most effective approaches to addressing difficult health-related issues is to seek collaboration with specialists from other disciplines, as is evident in the case of multidisciplinary research partnership^31^ ^,^ ^32^. Understanding of the viral genome has improved as a result of this all-encompassing approach, which also facilitates the application of these findings in actual disease prevention and control programs. There are various limitations to this study; however, given the frequent use of computational models and methods, their continuous development is necessary to maintain high accuracy and cross-genotype applicability. Furthermore, before these findings can be used in therapeutic settings, further experimental and clinical research is required to validate them, although this study provides interesting knowledge about the genome. By combining genomic data with other omics datasets, such as proteomics and transcriptomics, future research may better understand viral transmission patterns and host responses. 

The practical implementation of GRUs in real-world genomic analyses is essential for accurate and efficient outcomes. Challenges in applying GRU models to Genomic Datasets include data quality, computational complexity, model interpretability, and biological relevance. Genomic datasets are massive, high-dimensional, and often contain noise or missing values, making the application of GRU models challenging. GRUs are sensitive to noise, which significantly affects their performances. Cleaning, aligning, and normalizing sequences during preprocessing are essential for generating genomic data for the GRU models. GRUs perform well with sequential data. Biological sequences often have more complex patterns (nonlinear relationships, long-range dependencies, and domain-specific variations, such as epigenetic modifications) and may therefore require unique tuning of the encoding process. Improper handling of these aspects can result in inaccurate representations that do not correspond to actual genome sequences. The immense amount of genomic data is a significant challenge in GRU implementation. The computational challenge is extreme, with each human genome having approximately three billion base pairs, and considering whole populations or cross-species genomes further increases the computational burden. Training GRUs on large datasets requires significant computational memory, storage, and processing resources. Recurrent GRUs process sequences individually, which can be a bottleneck for long genomic sequences. This can be overcome by the efficient scaling of GRU models, such that terabytes and even petabytes of genomic data can be processed promptly. Data parallelism, distributed computing, and model optimization are essential for handling data size problems. However, these methods may only partially resolve the bottleneck in handling real-world genomic sizes. When applied to high-dimensional data, GRUs are susceptible to overfitting because the model memorizes the training data. Integrating domain-specific knowledge is essential to improve the biological relevance of GRU models in genome analysis. The application of GRUs in genomic analysis must address the practical challenges regarding data privacy and ethical considerations. Because GRU models operate with genomic data, the privacy and security of such data in cloud-based environments are priorities. Overcoming these limitations will necessitate the development of innovative algorithms, methodologies, and interdisciplinary work among data scientists, bioinformaticians, and biologists to create better-suited computational tools for genomic datasets.

## Data Availability

The entire dataset supporting the findings of this study is available upon request from the corresponding author, Abhishak Raj Devaraj. The dataset is not publicly available as research based on the current dataset is currently being continued.
